# Reconstructing Causal Biological Networks through Active Learning

**DOI:** 10.1371/journal.pone.0150611

**Published:** 2016-03-01

**Authors:** Hyunghoon Cho, Bonnie Berger, Jian Peng

**Affiliations:** 1 Computer Science and Artificial Intelligence Laboratory, MIT, Cambridge, MA, United States of America; 2 Department of Mathematics, MIT, Cambridge, MA, United States of America; 3 Department of Computer Science, University of Illinois at Urbana-Champaign, Champaign, IL, United States of America; University of Bonn, Bonn-Aachen International Center for IT, GERMANY

## Abstract

Reverse-engineering of biological networks is a central problem in systems biology. The use of intervention data, such as gene knockouts or knockdowns, is typically used for teasing apart causal relationships among genes. Under time or resource constraints, one needs to carefully choose which intervention experiments to carry out. Previous approaches for selecting most informative interventions have largely been focused on discrete Bayesian networks. However, continuous Bayesian networks are of great practical interest, especially in the study of complex biological systems and their quantitative properties. In this work, we present an efficient, information-theoretic active learning algorithm for Gaussian Bayesian networks (GBNs), which serve as important models for gene regulatory networks. In addition to providing linear-algebraic insights unique to GBNs, leading to significant runtime improvements, we demonstrate the effectiveness of our method on data simulated with GBNs and the DREAM4 network inference challenge data sets. Our method generally leads to faster recovery of underlying network structure and faster convergence to final distribution of confidence scores over candidate graph structures using the full data, in comparison to random selection of intervention experiments.

## Introduction

Molecules in a living cell interact with each other in a coordinated fashion to carry out important biological functions. Building a rich network of these interactions can greatly facilitate our understanding of human diseases by providing useful mechanistic interpretations of various phenotypes. Recent advances in high-throughput technologies have given rise to numerous algorithms for reverse-engineering interaction networks from molecular observations, as they provide an efficient and systematic way of analyzing the molecular state of a large number of genes. One class of such interaction networks that has generated much interest in recent years is transcriptional gene regulatory networks, which specify the set of genes that influence a given gene’s expression level. This type of pattern can be naturally modeled in a causal graph or Bayesian network.

Bayesian networks provide a compact way of representing causal relationships among random variables [1]. Given a directed acyclic graph (DAG) over the variables of interest, an edge *X* → *Y* encodes a causal influence of *X* on *Y*. However, when the given data consists of only passive observations of the underlying system, the causal structure is only identifiable up to Markov equivalence classes. To overcome this limitation, intervention experiments, in which some variables are controlled to take specific values, can be used to guarantee full identifiability given enough data [2]. For example, intervention on *X* only affects the behavior of *Y* in *X* → *Y*, but not in *X* ← *Y*; otherwise, if given only observational data for *X* and *Y*, these two graphs are indistinguishable. The importance of interventions for inferring biological networks has been noted in numerous studies [3–6]. In practical settings, interventions are typically performed via gene knockouts or knockdowns, i.e., by completely or partially reducing the expression level of one or more genes using experimental perturbations.

A key insight behind active learning is that not every variable is equally informative when intervened. For instance, if *X* does not have any children in every graph of a Markov equivalence class, perturbing *X* will not lead to any visible impact that can further distinguish the graphs. Thus, when the number of experiments that can be performed is limited, it is important to choose interventions which are most informative. In particular, it is generally not feasible to perform all possible interventions when joint interventions of multiple variables are considered.

Several researchers have developed active learning frameworks for causal structure learning during the last decade. In the Bayesian setting, Tong and Koller [7] and Murphy [8] both proposed decision-theoretic frameworks based on the expected reduction in uncertainty over edge directions and the expected change in posterior distribution over graph structures, respectively. While these approaches have been shown to be effective, they have been studied only in the context of discrete Bayesian networks. However, most molecular measurements are continuous, and hence they are more naturally described using continuous Bayesian networks. Based on this motivation, there have been a number of papers in the network inference literature which use Gaussian Bayesian networks (GBNs) as the underlying model, in which each variable is continuous and is modeled as a function of its parents with added Gaussian noise [9–12]. We contribute to this line of work by deriving the first Bayesian active learning algorithm for GBNs, where the informativeness of each candidate intervention is estimated via Bayesian inference, treating the graph as a latent random variable, and the most informative intervention is chosen. In the non-Bayesian setting, Hauser et al. [13], Eberhardt [2], and He and Geng [14] proposed active learning algorithms based on graph-theoretic insights, where the goal is to orient the most number of undirected edges in a Markov equivalence class with an intervention. Notably, these approaches aim only to determine the direction of edges in a given undirected graph (skeleton) estimated from observational data, and thus cannot handle errors already incorporated into the skeleton as a result of limited sample sizes and noisy observations. In this regard, our approach makes more effective use of intervention data by using it to improve the skeleton in addition to determining causal directions.

In this paper, we derive an efficient active learning algorithm for biological networks based on the framework of Murphy [8]. In addition to introducing an optimization technique unique to GBNs that leads to significant runtime improvement, we empirically validate the effectiveness of our algorithm on two data sets. Our results support the potential of active learning for uncovering casual structure in continuous-valued biological networks. Furthermore, our work enables researchers to effectively prioritize higher order joint perturbation experiments in a principled manner. This ability has the potential to accelerate the discovery of causal interactions between proteins, which are fundamental to advancing translational medicine and refining our understanding of biological systems.

## Methods

### Learning Gaussian Bayesian networks with interventions

#### Gaussian Bayesian networks

Let $X=\{X_{1},\cdots ,X_{n}\}$ be a set of random variables and G=(X,E) be a directed acyclic graph (DAG) over X, where (i,j)∈E if and only if there is a directed edge from *X*_*i*_ to *X*_*j*_. Let $Pa_{G}\left(j\right)=\left\{i|\left(i,j\right)\in E\right\}$ be the parent set of *X*_*j*_ in *G*. In a Gaussian Bayesian network (GBN), the conditional probability distribution (CPD) of each variable given the parents is defined to be a linear Gaussian distribution:

$$\begin{array}{c} X_{j}|X_{Pa_{G}\left(j\right)}∼N(m_{j}+\sum_{i\in Pa_{G}\left(j\right)}w_{ij}X_{i},\;\sigma_{j}^{2}) \end{array}$$

where *X*_*S*_ := {*X*_*i*_}_*i* ∈ *S*_. Note *m*_*j*_ and $\sigma_{j}^{2}$ represent the base level and conditional variance of *X*_*j*_, respectively, and *w*_*ij*_ represents the weight of causal effect along the edge (*i*, *j*). For compactness, we denote the set of parameters {*m*_*j*_}, {*w*_*ij*_}, and $\left\{\sigma_{j}^{2}\right\}$ for a particular graph *G* as Θ^*G*^. A GBN model *M* = (*G*, Θ^*G*^) fully defines a joint probability density function (PDF) over X as a product of Gaussian PDFs, and the set of independence assumptions that the joint PDF satisfies is encoded in the structure *G*. Furthermore, it can be shown that the joint PDF defined by *M* is, in fact, multivariate normal.

#### Structure learning with observational and intervention data

A standard approach to inferring Bayesian network structure from data involves defining a score that reflects how well a given graph explains the data and searching for high-scoring graphs in the space of DAGs or causal node orderings. Typically, a Markov chain Monte Carlo (MCMC) method based on random walks is used to explore the space of candidate graph structures and to select the highest-scoring graph structure. In this section, we describe a Bayesian scoring function, which evaluates the posterior probability of a structure given the data. This scoring function constitutes an important component of the active learning algorithm we will develop next.

Given an instance of *observational* data where every variable is observed, ***x*** = (*x*_1_, …, *x*_*n*_), the likelihood *p*(***x***|*G*, Θ^*G*^) of a GBN model *M* = (*G*, Θ^*G*^) can be expressed as

$$\begin{array}{c} \prod_{j=1}^{n}N(x_{j};m_{j}+\sum_{i\in Pa_{G}\left(j\right)}w_{ij}x_{i},\;\sigma_{j}^{2}), \end{array}$$ (1)

where *N*(⋅; *μ*, *σ*^2^) is the normal PDF with mean *μ* and variance *σ*^2^.

Under an intervention (e.g., gene knockout or RNAi), a subset of random variables in X are clamped at specific values and the remaining variables are assumed to be jointly sampled from a modified graph where the incoming edges of the clamped nodes are removed. Intuitively, this ensures that the nodes *upstream* of the clamped nodes are unaffected by the intervention. Let *I* denote the setup of an intervention experiment and ***x*** = (*x*_1_, …, *x*_*n*_) be the outcome. For each (*i*, *c*_*i*_) ∈ *I*, the value of *X*_*i*_ is clamped at a constant *c*_*i*_ (i.e., *x*_*i*_ = *c*_*i*_). The likelihood function *p*(***x***|*I*, *G*, Θ^*G*^) for an intervention data instance (***x***, *I*) is given by

$$\begin{array}{c} \prod_{(j,\cdot )\notin I}N(x_{j};m_{j}+\sum_{i\in Pa_{G}\left(j\right)}w_{ij}x_{i},\;\sigma_{j}^{2}). \end{array}$$ (2)

The only difference from the observational case is that the product is now only over the nodes that are not clamped. When no variables are clamped (*I* = ∅), the above expression is consistent with Eq (1).

Now, let *D* be a sequence of *m* data instances, ***x***^(1)^, …, ***x***^(*m*)^, and I be the sequence of corresponding experimental setups, *I*^(1)^, …, *I*^(*m*)^. This can be viewed as a collection of both observational (*I* = ∅) and intervention (*I* ≠ ∅) experiments. The *complete likelihood function* $p(D|I,G,\Theta^{G})$ for the data set is given by

$$\begin{array}{c} \prod_{k=1}^{m}\prod_{\left(j,\cdot \right)\notin I^{\left(k\right)}}N(x_{j}^{\left(k\right)};m_{j}+\sum_{i\in Pa_{G}\left(j\right)}w_{ij}x_{i}^{\left(k\right)},\;\sigma_{j}^{2}). \end{array}$$

By arranging terms for each family (i.e., a node and its parents) across data instances, this can be rewritten as

$$\begin{array}{c} \prod_{j=1}^{n}\prod_{k:\left(j,\cdot \right)\notin I^{\left(k\right)}}N(x_{j}^{\left(k\right)};m_{j}+\sum_{i\in Pa_{G}\left(j\right)}w_{ij}x_{i}^{\left(k\right)},\;\sigma_{j}^{2}). \end{array}$$

The fact that the likelihood over intervention data still decomposes into family-specific terms (each over a mutually exclusive set of parameters) enables the use of a conjugate prior similar to the one introduced by Geiger and Heckerman [15] that gives us a closed-form expression for the posterior. Here we impose an *independent* normal-inverse Gamma prior over each set of family-specific parameters, $\Theta_{j}^{G}$, which consists of *m*_*j*_, {*w*_*ij*_}_*i* ∈ *Pa*_*G*_(*j*)_, and $\sigma_{j}^{2}$. An advantage of this representation is that we are now able to compute the complete posterior scoring function by simply calculating the posterior for each family and multiplying them together.

Specifically, for each node *j*, let ***θ***_*j*_ be a column vector (*m*_*j*_, *w*_*p*_1_*j*_, …, *w*_*p*_*d*_*j*_) where *p*_1_, …, *p*_*d*_ is an enumeration of elements in *Pa*_*G*_(*j*). Let *k*_1_, …, *k*_*t*_ be an enumeration of {*k* : *j* ∉ *I*^(*k*)^} (i.e., instances where *X*_*j*_ is not clamped). We define a *family-specific data set* (***X***_*j*_, ***y***_*j*_) for node *j* as

$$\begin{array}{c} X_{j}=[\begin{array}{cccc} 1 & x_{p_{1}}^{\left(k_{1}\right)} & \cdots & x_{p_{d}}^{\left(k_{1}\right)}\\ \vdots & \vdots & \ddots & \vdots \\ 1 & x_{p_{1}}^{\left(k_{t}\right)} & \cdots & x_{p_{d}}^{\left(k_{t}\right)} \end{array}],\;y_{j}=[\begin{array}{c} x_{j}^{\left(k_{1}\right)}\\ \vdots \\ x_{j}^{\left(k_{t}\right)} \end{array}], \end{array}$$

which depends on *G*, *D*, and I. Now, if we assume the following prior distribution for $\Theta_{j}^{G}$:

$$\begin{array}{ccc} \sigma_{j}^{2} & ∼ & \text{Inv-Gamma}(\alpha_{j},\beta_{j})\\ \theta_{j}|\sigma_{j}^{2} & ∼ & N(\mu_{j},\sigma_{j}^{2}{\left(\Lambda_{j}\right)}^{-1}) \end{array}$$

with hyperparameters *α*_*j*_, *β*_*j*_, ***μ***_*j*_, and **Λ**_*j*_, then the posterior distribution $p(\Theta_{j}^{G}|D,I,G)$ has the same form as the prior, with the following updated parameters:

$$\begin{array}{c} \Lambda_{j}^{\prime }:=X_{j}^{T}X_{j}+\Lambda_{j}, \end{array}$$ (3)

$$\begin{array}{c} \mu_{j}^{\prime }:={\left(\Lambda_{j}^{\prime }\right)}^{-1}\left(\Lambda_{j}\mu_{j}+X_{j}^{T}y_{j}\right), \end{array}$$ (4)

$$\begin{array}{c} \alpha_{j}^{\prime }:=\alpha_{j}+\frac{\left|D\right|}{2}, \end{array}$$ (5)

$$\begin{array}{c} \beta_{j}^{\prime }:=\beta_{j}+\frac{1}{2}(y_{j}^{T}y_{j}+\mu_{j}^{T}\Lambda_{j}\mu_{j}-{\left(\mu_{j}^{\prime }\right)}^{T}\Lambda_{j}^{\prime }\mu_{j}^{\prime }). \end{array}$$ (6)

Moreover, the *marginal likelihood function* p(D|I,G), which usually requires a challenging step of integrating out the model parameters Θ^*G*^ to compute, can now be analytically obtained as

$$\begin{array}{c} {\left(2\pi \right)}^{-c(D,I)/2}\prod_{j=1}^{n}\sqrt{\frac{\det(\Lambda_{j})}{\det(\Lambda_{j}^{\prime })}}\cdot \frac{{\left(\beta_{j}\right)}^{\alpha_{j}}}{{\left(\beta_{j}^{\prime }\right)}^{\alpha_{j}^{\prime }}}\cdot \frac{\Gamma (\alpha_{j}^{\prime })}{\Gamma (\alpha_{j})}, \end{array}$$ (7)

where $c\left(D,I\right)=n|D|-\sum_{I\in I}\left|I\right|$ is the sum of the number of unclamped variables in each data instance.

Since p(G|D,I)∝p(D|I,G)p(G), given the analytical expression for marginal likelihood, one can explore the posterior distribution over the space of candidate graph structures using the Metropolis-Hastings (MH) algorithm [16, 17]. Unfortunately, an in-depth discussion of different ways in which one can set up various components of this procedure, including the design of search space, prior over graphs, and proposal distribution, is out of the scope of this paper. The output of this algorithm is a set of sampled graph structures drawn from the posterior p(G|D,I), which intuitively represents how strongly we believe each candidate graph structure to be the underlying model for the given data. This output can be summarized in a number of ways to construct the finalized model. The most common approach is to employ *Bayesian model averaging*, in which a feature of interest *f* (e.g., presence of edge) is averaged over all graph samples to obtain E[f|D,I].

### Prioritizing interventions via active learning

Most network inference methods, including the one presented in the previous section, assume that the data set is obtained and fixed prior to learning. However, in a real world setting, one can perform additional intervention experiments and combine them with existing data to improve the quality of learned networks. An active learning framework allows us to reason about how *informative* each candidate experiment is, thus enabling a more efficient design of intervention experiments when subjected to time or resource constraints.

Here, we present our active learning algorithm for inferring the structure of GBNs. We adopt the information-theoretic framework developed by Murphy [8] and introduce an optimization based on linear-algebraic insights unique to GBNs which serve to improve the overall complexity of the algorithm over a naive implementation.

#### Greedy selection

Let C be the set of candidate intervention experiments. Following Murphy [8], we define the *I*^⋆^ to be the optimal experiment which maximizes the *mutual information* (MI) between the resultant outcome X and *G*, given the current data set (D,I). In other words,

$$\begin{array}{c} I^{⋆}={\arg\max}_{I\in C}\psi \left(I\right), \end{array}$$

where the objective function ψ(I):=MI(G;X|D,I) can be alternatively expressed in two different ways as

$$\begin{array}{c} E_{X∼p(\cdot |I,D,I)}\left[\text{KL}\left(p\left(G|X,I,D,I\right)\;∥\;p\left(G|D,I\right)\right)\right] \end{array}$$ (8)

and

$$\begin{array}{c} E_{G∼p(\cdot |D,I)}\left[\text{KL}\left(p\left(X|G,I,D,I\right)\;∥\;p\left(X|I,D,I\right)\right)\right]. \end{array}$$ (9)

KL(⋅‖⋅) denotes the Kullback-Leibler divergence. Eq (8) provides a useful insight that the optimal intervention is the one that is expected to cause the largest change (measured by divergence) in our belief over the candidate graph structures. On the other hand, Eq (9) turns out to be easier to compute. In particular, based on Eq (9), *ψ*(*I*) can be expressed as

$$\begin{array}{c} E_{G∼p(\cdot |D,I)}[E_{X∼p(\cdot |G,I,D,I)}[\Delta (G,X)]], \end{array}$$ (10)

where

$$\begin{array}{c} \Delta \left(G,X\right)=\log(\frac{p(X|G,I,D,I)}{E_{G∼p(\cdot |D,I)}[p(X|G,I,D,I)]}). \end{array}$$ (11)

Apart from the expectations, the only term that needs to be evaluated is the marginal likelihood p(X|G,I,D,I), for which we have an analytical expression as given in Eq (7) (with p(G|D,I) as the new prior).

Computing expectations over *G* and X are both intractable, so we replace them with approximations based on random samples. Let *G*_1_, …, *G*_*S*_ be random samples from the posterior distribution p(G|D,I), which can be obtained using an MCMC method as previously described. To avoid drawing separate samples of X for each graph sample for computational reasons, we use importance sampling for the inner expectation over X with a sampling distribution q(X|I,D,I) that is independent of *G*. In our experiments, we used $q:=p(X|G^{\circ },I,D,I)$ where *G*^∘^ is the graph with no edges where every variable is independent. Letting ***x***_1_, …, ***x***_*R*_ be random samples from *q*, Eq (10) can be approximated as

$$\begin{array}{c} \frac{1}{S}\sum_{s=1}^{S}\sum_{r=1}^{R}v_{rs}\log(\frac{p(x_{r}|I,G_{s},D,I)}{\frac{1}{S}\sum_{s^{\prime }=1}^{S}p\left(x_{r}|I,G_{s^{\prime }},D,I\right)}), \end{array}$$ (12)

where $v_{rs}={\tilde{v}}_{rs}/\sum_{r^{\prime }=1}^{R}{\tilde{v}}_{r^{\prime }s}$ with

$$\begin{array}{c} {\tilde{v}}_{rs}:=\frac{p(x_{r}|I,G_{s},D,I)}{q(x_{r}|I,D,I)}. \end{array}$$

**Fig 1:**
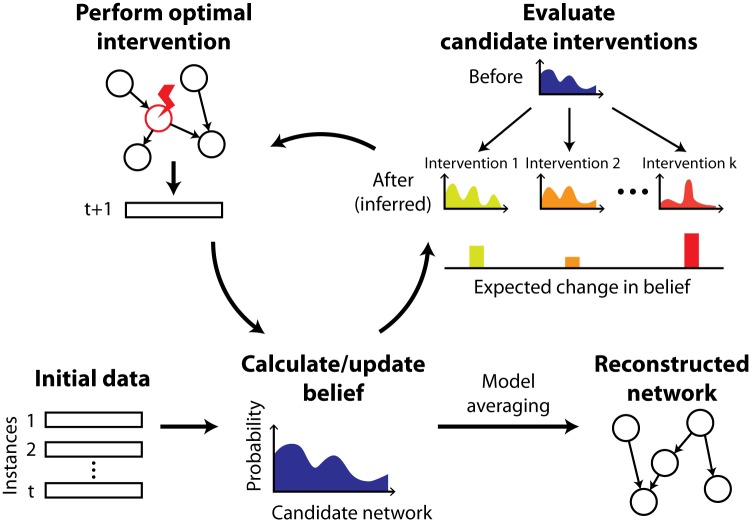
Active learning framework for network reconstruction. We first estimate our belief over candidate graph structures based on the initial data set that contains observational and/or intervention samples. Then, we iteratively acquire new data instances by carrying out the optimal intervention experiment predicted to cause the largest change in our belief (in expectation) and updating the belief. The final belief is summarized into a predicted network via Bayesian model averaging.

The overall active learning procedure, with the optimization technique discussed in the following section, is outlined in Algorithm 1 and Fig 1. We provide a MATLAB implementation of our algorithm in S1 Code.

**Algorithm 1** Active learning for GBN

**Require**: Candidate graph structures G, prior over graphs *p*(*G*), initial data set $\left(D^{\left(0\right)},I^{\left(0\right)}\right)$, candidate interventions C, number of nodes *n*, number of additional experiments to perform *T*, number of graph samples *S*, number of samples for experimental outcome *R*

 Sample $G_{1}^{\left(0\right)},\cdots ,G_{S}^{\left(0\right)}∼p\left(G|D^{\left(0\right)},I^{\left(0\right)}\right)$ via MCMC

 **for** *t* = 1 **to** *T* **do**

  **for all** I∈C **do**

   Sample ***x***_1_, …, ***x***_*R*_ from $q(x|I,D^{\left(t-1\right)},I^{\left(t-1\right)})$

   **for** *s* = 1 **to** *S* **do**

    **for** *j* = 1 **to** *n* **do**

     Using Eqs (3)–(6), compute *α*_*j*_, *β*_*j*_, ***μ***_*j*_, and **Λ**_*j*_ of $p(\Theta_{j}^{G_{s}^{\left(t-1\right)}}|D^{\left(t-1\right)},I^{\left(t-1\right)},G_{s}^{\left(t-1\right)})$

     Compute $\Lambda_{j}^{-1}$ and det(**Λ**_*j*_)

    **end for**

    **for** *r* = 1 **to** *R* **do**

     Using Eqs (7), (13) and (14), compute $p(x_{r}|I,G_{s},D^{\left(t-1\right)},I^{\left(t-1\right)})$

    **end for**

   **end for**

   Using Eq (12), estimate *ψ*(*I*)

  **end for**

  $I^{⋆}⇐{\arg\;\max}_{I\in C}\psi \left(I\right)$

  Perform experiment under *I*^⋆^, record the outcome ***x***

  *D*^(*t*)^ ⇐ (*D*^(*t*−1)^,***x***), $I^{\left(t\right)}⇐\left(I^{\left(t-1\right)},I^{⋆}\right)$

  Sample $G_{1}^{\left(t\right)},\cdots ,G_{S}^{\left(t\right)}∼p\left(G|D^{\left(t\right)},I^{\left(t\right)}\right)$ via MCMC, initialize with $G_{1}^{\left(t-1\right)},\cdots ,G_{S}^{\left(t-1\right)}$

 **end for**

 **return** averaged model of $G_{1}^{\left(T\right)},\cdots ,G_{S}^{\left(T\right)}$

#### Efficient calculation of marginal likelihood

The computational bottleneck of our algorithm is in the evaluation of $p(x_{r}|I,G_{s},D,I)$ for every combination of *I*, *r*, and *s*. This involves calculating the posterior parameters for *G*_*s*_ given (D,I) and also the updated posterior after observing (***x***_*r*_, *I*). The former need only be computed once for each *G*_*s*_. For the latter, the fact that only a single instance is added to the data set allows a more efficient computation of ${\left(\Lambda_{j}^{\prime }\right)}^{-1}$ in Eq (4) and $\det(\Lambda_{j}^{\prime })$ in Eq (7). In particular, an application of the Sherman-Morrison formula and the matrix determinant lemma gives us:

$$\begin{array}{ccc} {\left(\Lambda_{j}^{\prime }\right)}^{-1} & = & {\left(\Lambda_{j}+vv^{T}\right)}^{-1}\\ & = & \Lambda_{j}^{-1}-\frac{\Lambda_{j}^{-1}vv^{T}\Lambda_{j}^{-1}}{1+v^{T}\Lambda_{j}^{-1}v}, \end{array}$$ (13)

$$\begin{array}{ccc} \det(\Lambda_{j}^{\prime }) & = & \det(\Lambda_{j}+vv^{T})\\ & = & \left(1+v^{T}\Lambda_{j}^{-1}v\right)\det\left(\Lambda_{j}\right), \end{array}$$ (14)

where *v*^*T*^ is the row of the family-specific data set ***X***_*j*_ that corresponds to the new outcome ***x***_*r*_. Essentially, by saving the inverse and determinant of **Λ**_*j*_ for each *G*_*s*_, one can reduce the compute time of $p(x_{r}|I,G_{s},D,I)$ from *O*(*md*^2^) to *O*(*d*^2^) where *m* is the number of samples in the data and *d* is the upper bound on the number of parents each node can take.

#### Evaluation of network reconstruction performance

We assessed the performance of our learning algorithm in several different ways. To analyze how accurately we learned the underlying causal structure, we followed the evaluation scheme used in the DREAM4 challenge [18] and calculated the area under receiver operating characteristic curve (AUROC) and the area under precision recall curve (AUPRC) based on a ranked list of edges. The absolute value of the expected maximum a posteriori (MAP) edge weight $E[{\hat{w}}_{ij}^{\text{MAP}}|D,I]$, approximated using graph samples from the posterior, was used as the score for each edge. On our simulated data, we also calculated the mean-squared error (MSE) of the expected MAP edge weights (over *n*(*n* − 1) possible edges) since we have access to the true parameters.

In addition to analyzing the trajectory of different accuracy measures over the course of the iterative learning procedure where one intervention experiment is added at a time, we also looked at a metric that is agnostic to whether we have access to the ground truth network. When we are given a data set with pre-generated interventions and their outcomes, we can retroactively evaluate, given any subset of the data set, how close we are to the final belief over candidate graph structures obtained using the whole data set. The final belief is expected to better reflect the ground truth, and thus faster convergence to the final belief is desirable in most cases. Intuitively, this evaluates how much information we lose if we only had enough resources to perform a small subset of the intervention experiments provided. We measure this by calculating the KL divergence of the final belief from the current belief over 5000 randomly chosen candidate graphs.

## Results

### GBNs can capture causal relationships in biological data

**Fig 2:**
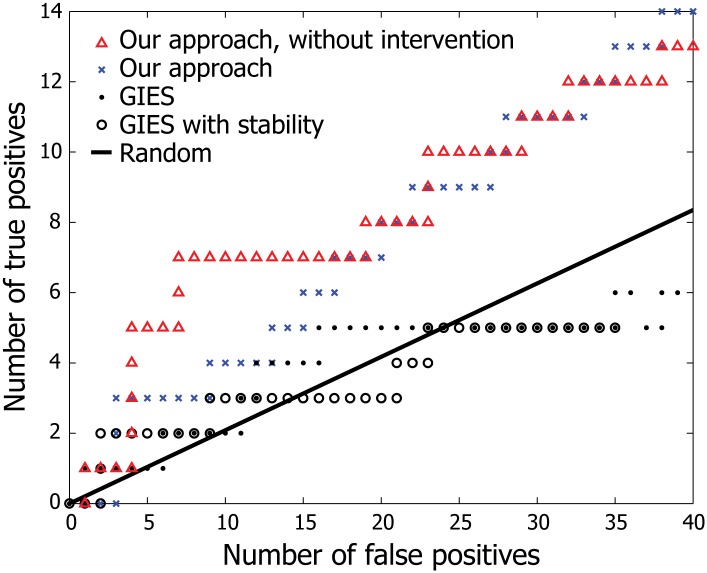
Reconstruction performance on single cell gene expression data. We applied our Bayesian structure learning algorithm based on GBNs to uncover the signaling pathway of 11 human proteins from expression data provided by Sachs et al. [5]. MAP estimates of edge weights calculated using 1,000 posterior graph samples are used to generate a ranked list of (directed) edges for evaluation of accuracy. The data points for GIES are taken from Hauser and Bühlmann [19] for comparison. The result suggests GBNs can uncover causal edges in real biological networks, and that our approach is more effective than GIES.

We first set out to test whether the model assumptions of GBNs (acyclicity and Gaussianity) are too restrictive to be effectively applied to real biological data. We ran our algorithm on gene expression data collected by Sachs et al. [5], which consists of 7,466 single cell expression profiles of 11 phosphorylated proteins involved in a signaling pathway of human primary T cells. A subset of measurements were taken from cells under perturbation induced by different reagents that activate/inhibit a particular protein in the pathway. We applied the same Bayesian structure learning algorithm for GBNs used in our framework to recover the ground truth signaling pathway (adopted from Sachs et al. [5]), and were able to predict causal links among the proteins with reasonable accuracy (0.65 AUROC and 0.30 AUPRC, averaged across five runs of MCMC). This shows that GBNs can detect edges in a real network despite the model assumptions. In addition, our inference algorithm outperformed GIES, a state-of-the-art non-Bayesian approach [19] for learning GBNs, providing further support for our Bayesian learning approach (Fig 2). Notably, the inclusion of intervention samples did not improve prediction accuracy on this data set. As previously pointed out by Mooij et al. [20], this odd behavior is likely due to the fact that the experimental perturbation employed by Sachs et al. [5] modifies the *activity* of the target protein instead of its *abundance*, which is the intended setting of our method. It is worth noting that, while Sachs et al. [5] reconstructs the ground truth network with greater accuracy, this is likely dependent on a carefully chosen discretization of the input data [19, 20], which is precisely the type of tuning we aim to avoid by using *continuous* Bayesian networks.

### Active learning accelerates network reconstruction on simulated data

**Fig 3:**
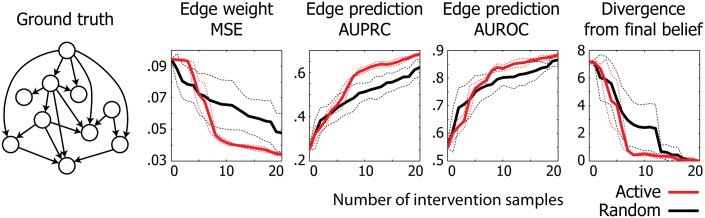
Reconstruction performance on simulated data from a GBN. We compared edge prediction performance between active and random learners, summarized over five trials. The dotted lines are drawn at one standard deviation from the mean in each direction. Active learner achieves higher accuracy and faster convergence than random learner.

To demonstrate the effectiveness of our active learning algorithm, we randomly generated a GBN with 10 nodes (Fig 3) as ground truth and generated a collection of observational and intervention samples from the model. Given this simulated data, we set out to compare the reconstruction performance of an active learner with that of a random learner, which selects intervention experiments uniformly at random.

The parameters of the ground truth GBN are generated as follows. Each edge weight *w*_*ij*_ is uniformly sampled from (−1,−.25) ∪ (.25,1). The base level *m*_*j*_ of each node is sampled from N(0,1), and the noise level *σ*_*j*_ is set to 0.05 for all nodes. After populating the parameters, we sampled 10 observational instances to be used as the initial data set and ran both active and random learners until they iteratively selected 20 additional intervention experiments. Here, we only consider single variable knockout (clamping at zero) as possible interventions. For the convergence analysis, two instances of each knockout were pre-generated and the learners were limited to using them without replacement.

For the MH algorithm used for sampling graphs from the posterior distribution at each iteration, we used a proposal distribution that assigns uniform weight to each DAG in the neighborhood that is reachable by a single-edge insertion, deletion, or reversal, following the suggestions of Giudici et al. [21]. Also, *p*(*G*) was set to be uniform over DAGs with maximum in-degree of five; imposing a limit on the number of parents is a commonly used heuristic in the literature [22]. On the initial data set, we used a burn-in of 10,000 steps and thinning of 100 steps to obtain the first batch of graph samples. For the subsequent belief updates, we propagated each graph sample by 100 steps to obtain the new batch. Note that the change in posterior distribution after each iteration is relatively small because only one additional data instance is added. We used 1,000 graph samples and 100 experimental outcome samples (i.e., *S* = 1000, *R* = 100).

The results are summarized in Fig 3. We observe that our active learning algorithm achieves consistently higher accuracy than random learner across all three metrics (MSE, AUPRC, AUROC) after the first few iterations, leading to higher final accuracy overall. We also observe a faster convergence rate for our method. In particular, our algorithm achieved a belief that is close (divergence < 1) to the final belief after seven interventions, while random learner reached the same level only after almost twice as many interventions.

### Active learning accelerates network reconstruction on DREAM4 benchmark data

We next asked whether we can achieve a similar improvement on a data set that more closely resembles biological data. To this end, we tested our method on data from the DREAM4 10-node in-silico network reconstruction challenge [18], which is a commonly used benchmark data for network inference algorithms. They provide five networks with different structures, all chosen to reflect common topological properties of real gene regulatory networks in *E. coli* or *S. cerevisiae*, which include feedback loops. Stochastic differential equations and a realistic noise model of microarray data sets are used to generate expression data from each network. We jointly considered the wild type and 10 multifactorial perturbation data as the initial observational data set (11 instances total), and ran active and random learners to prioritize 20 intervention samples, which consist of one knockout and one knockdown per gene. We made a simplifying assumption that the learner knows the resulting expression level of the target gene in a knockdown experiment. It is straightforward to properly address this uncertainty in a practical setting by taking the expectation with respect to the target variable using a sampling approach.

**Fig 4:**
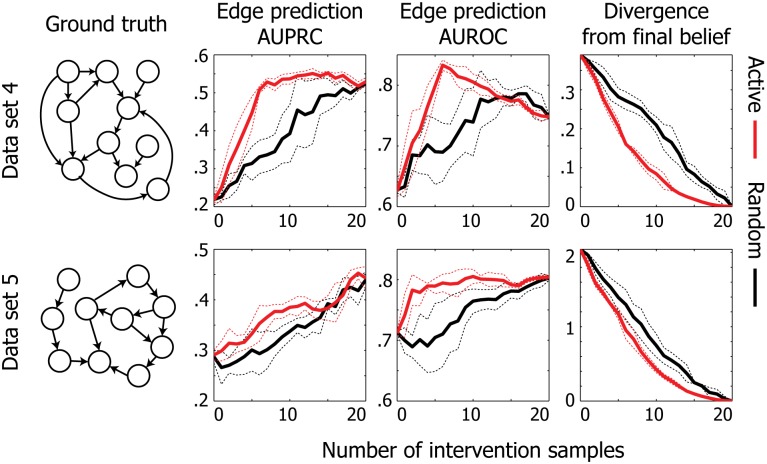
Reconstruction performance on DREAM4 benchmark data. The results are summarized over five trials. The dotted lines are drawn at one standard deviation from the mean in each direction. Active learner achieves higher accuracy and faster convergence than random learner.

The results from the DREAM4 analysis are summarized in Fig 4. Since our method is based on acyclic graphs, we focused our analysis on data sets 4 and 5, which are generated from networks that contain fewer and weaker (i.e., longer) cycles than the remaining data sets. We observe a clear performance improvement by our active learning algorithm in terms of the speed at which we recover the underlying causal structure. Furthermore, the convergence rate of our method was consistently and significantly faster on both data sets. Note that the final accuracy of our method is comparable to earlier work that also applied GBNs to analyze the DREAM4 data set [12]. The results on data sets 1–3 along with their ground truth networks are provided in S1 Fig. In the case where the model assumption is heavily violated (i.e., there are relatively numerous and short cycles), our method still achieves significantly faster convergence to the final belief. However, due to the cyclic nature of these data sets, our method achieves generally lower final accuracies on these data sets and does not show a clear improvement over the random learner.

**Fig 5:**
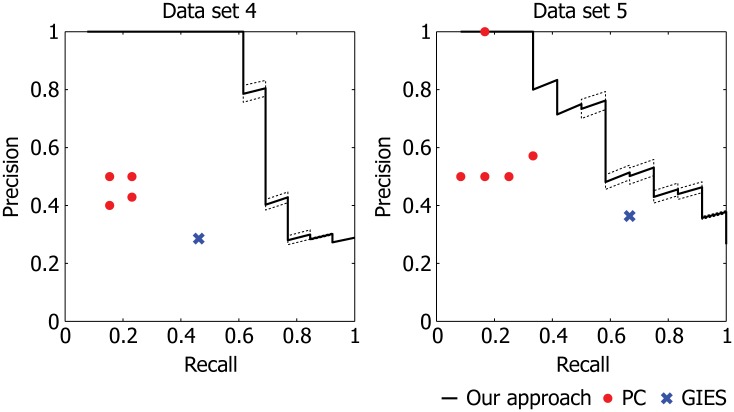
Performance comparison with PC and GIES on DREAM4 data sets. We evaluated the final prediction accuracy of our active learning algorithm in identifying edges in the undirected skeleton of the ground truth network. The resulting precision-recall (PR) curves were compared to PC with different values of *α* (significance level) in {0.01, 0.05, 0.1, 0.2, 0.3} using only observational data and to GIES using both observational and intervention data. We used the implementations of PC and GIES provided in the pcalg package in R. The dashed lines are drawn at one standard deviation from the mean in each direction based on five random trials. Our performance generally dominates that of PC and GIES, suggesting the effectiveness of our Bayesian learning approach.

He and Geng [14] previously proposed an algorithm that prioritizes interventions to maximally orient the edges with ambiguous direction in a Markov-equivalence class, estimated by a standard network inference algorithm, PC [23]. Given enough observational samples, the PC algorithm recovers the graph structure up to Markov-equivalence based on conditional independence tests. We wish to emphasize that our Bayesian inference framework, unlike He and Geng’s approach, takes advantage of intervention samples not only for determining edge directions but also for refining the undirected skeleton of the graph. Such an approach is essential in a practical setting where the observational data is limited in both quantity and quality, which can lead to numerous incorrect or missing edges in the skeleton. We empirically observed in the DREAM4 data sets that our active learning method predicts the ground truth skeleton with higher accuracy than PC (Fig 5). Moreover, our method outperformed GIES [19] when applied to the full DREAM4 data (without prioritization). GIES employs a greedy search over candidate graphs taking both observational and intervention data into account. These results suggest that our learning approach more effectively uncovers the true graph structure than other methods developed for network inference based on intervention data.

**Fig 6:**
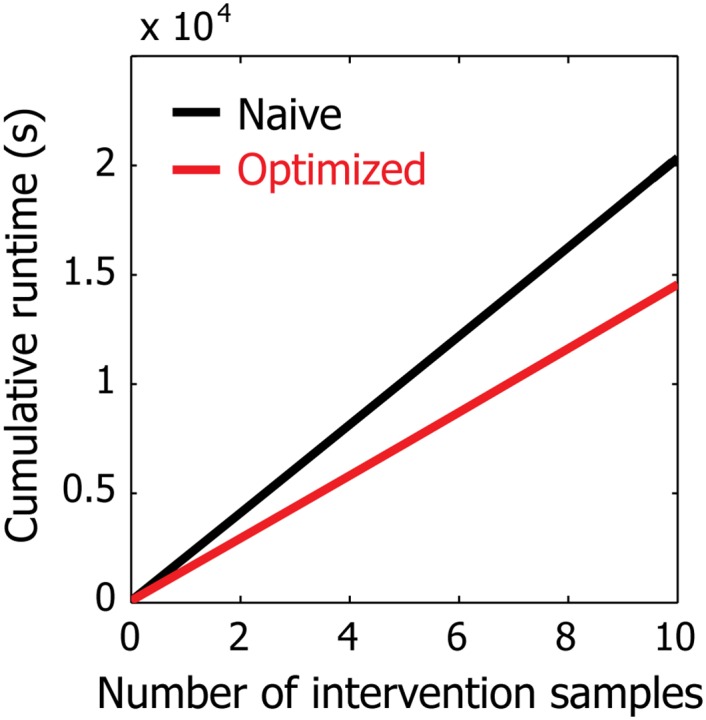
Runtime improvement of our method on simulated data. The results are summarized over three trials (error bands are not visible due to low variance). Our optimization technique specific to GBNs leads to significant improvement in runtime.

Lastly, we tested the extent to which our optimization based on rank-one updates to the matrix inverse and determinant improves the runtime of our algorithm. The cumulative runtime of the iterative learning procedure on our simulated data is shown in Fig 6. Overall, our optimization is accountable for ∼30% reduction in runtime. We expect the improvement to be even more significant on data sets with more samples. Note that this analysis was conducted using a single 3.47 GHz Intel Xeon X5690 CPU for fairness of comparison even though our algorithm easily lends itself to parallelism and one can obtain significantly faster runtimes with multiple CPUs. However, despite our runtime improvement, we note that our method is currently intended only for small-scale networks (e.g., <30 nodes), as is the case for most Bayesian network inference algorithms due to the super-exponential growth of the number of candidate graphs with respect to the number of nodes. We expect our method to be most effective for studies where practitioners aim to tease apart causal influences among a small set of genes or proteins of interest, such as a group of genes that belong to a specific biological process.

## Discussion

In this paper, we derived an efficient active learning algorithm for Gaussian Bayesian networks and demonstrated its effectiveness on several data sets. We showed that our algorithm achieves a clear improvement in uncovering the true network as long as the underlying causal structure does not significantly violate the acyclicity assumption inherent in the GBN models. Even under violation of model assumption, we were able to observe superior convergence rate of the active learner, which further supports the effectiveness of our method.

There are several important ways in which this work could be improved for better applicability in systems biology in the future. First, we could develop a systematic way of selecting a batch of intervention experiments to be performed simultaneously, which is a more suitable setup for high-throughput assays. Second, we could further adopt our method to support perturbation experiments in which we only observe the response of a single reporter gene, whose phenotype (e.g., luminescence) is easier to quantify than systematic expression profiling. Third, it would be interesting to look for better ways to find optimal intervention other than exhaustive enumeration followed by linear search for the optimal solution. This capability is especially of interest as we consider higher-order interventions of multiple variables, in order to counter the combinatorial explosion in the number of candidate interventions to consider.

## Supporting Information

S1 Fig Reconstruction performance on DREAM4 data sets 1–3. Even when the ground truth network contains numerous short cycles, our method still achieves significantly faster convergence to the final belief. However, due to the violation of model assumption, our method achieves generally lower final accuracies than those of data sets 4 and 5 and does not clearly outperform random learner. The results are summarized over five trials. The dotted lines are drawn at one standard deviation from the mean in each direction.
(EPS)

S1 Code MATLAB implementation of our algorithm with an example data set. (ZIP)
